# In vivo experimental study on the resistance and stiffness of orbital suspension tissues with/without the extraocular muscles

**DOI:** 10.1186/s12938-019-0688-4

**Published:** 2019-05-31

**Authors:** Hongmei Guo, Zhipeng Gao, Baoyan Han, Lijun Zhang, Zhaoqiang Tang, Jing Chen, Lili Wang, Weiyi Chen

**Affiliations:** 1 Shanxi Key Laboratory of Material Strength & Structural Impact, College of Biomedical Engineering, Taiyuan University of Technology, 79 YingZe West Street, Taiyuan, 030024 Shanxi China; 2 Department of Ophthalmology, Bethune International Peace Hospital of PLA, Shijiazhuang, 050082 Hebei China; 3 Department of Strabismus and Pediatric Ophthalmology, Shanxi Eye Hospital, Taiyuan, 030002 Shanxi China

**Keywords:** Orbital suspension tissue, Resistance, Stiffness, Rabbit

## Abstract

**Background:**

The accuracy of the surgical amount of extraocular muscle (EOM) is key to the success of strabismus surgery. To establish an accurate eye movement model, it is of great theoretical value and clinical significance to determine the surgical amount of EOM. At present, only resistance and stiffness data of orbital suspension tissues with EOMs exist, while those of orbital suspension tissues without EOMs, which is critical information for eye movement modeling, have not been reported. The aim of this research is to study the resistance and stiffness of orbital suspension tissues with/without EOMs.

**Methods:**

Fifteen healthy New Zealand white rabbits with body weights of 2.41 ± 0.13 kg were used in the study. Two recti (two horizontal recti of the left eye or two vertical recti of the right eye) or all EOMs were detached from each eye under general anesthesia. Then, a 5-0 silk suture was attached to the stump of the detached rectus insertion (two horizontal recti insertions of the left eye and two vertical recti insertions of the right eye) on the isolated eyeball. The 5-0 silk suture was connected to the INSTRON 5544 tester to facilitate the horizontal rotations of the left eyes and the vertical rotations of the right eyes, respectively.

**Results:**

The resistance and stiffness of orbital suspension tissues with superior rectus, inferior rectus, superior oblique, and inferior oblique EOMs were obtained during horizontal eye movement. Similarly, the resistance and stiffness of orbital suspension tissues with lateral rectus, medial rectus, superior oblique, and inferior oblique EOMs were obtained during vertical eye movement. Then, the resistance and stiffness of orbital suspension tissues without EOMs were obtained during horizontal and vertical eye movements. The resistance and stiffness data of orbital suspension tissues with EOMs were compared with those of orbital suspension tissues without EOMs. The comparison results showed no significant difference in the resistance values between these two cases. In addition, the stiffness values of these two cases statistically differed.

**Conclusions:**

The two horizontal recti play a major role in passive horizontal eye movement. In addition, when the eye is passively moved vertically, the two vertical recti play major roles. The stiffness of orbital suspension tissues with EOMs, which has been used in eye movement modeling, is not accurate. The results of this work may serve as a reference for improving the accuracy in eye movement modeling, and then it will be beneficial for determining the surgical amount of EOMs in clinical surgery.

## Introduction

Six extraocular muscles (EOMs) and peripheral orbital suspension tissues control eye movement. The six EOMs include the lateral rectus (LR), medial rectus (MR), superior rectus (SR), inferior rectus (IR), superior oblique (SO), and inferior oblique (IO). Orbital suspension tissues or passive orbital tissues include all nonmuscular suspensory tissues, such as Tenon’s capsule, the optic nerve, the fat pad, and the conjunctive [1]. The resistance and stiffness of orbital suspension tissues are essential for eye movement modeling [2]. The normal life of humans is seriously affected by eye movement disorders, in which only the incidence of the strabismus is up to 3–4% [3]. The treatment of strabismus is based on EOM surgery. In addition, the accuracy of the surgical amount of EOMs is the key to successful EOM surgery. At present, ophthalmologists generally used their own experience to determine the surgical amount of EOMs [4]. This method lacks unified guiding standards, and the accuracy of the operation is difficult to master. An eye movement model determining the surgical amount of EOMs has great theoretical value and clinical significance for the individualized design of the surgical amount of EOM diseases, such as strabismus. The precise eye movement model can not only provide accurate theoretical guidance to increase the precision of the clinical EOM surgical amount but also promotes the development of ocular prosthesis, a humanoid robot, and other modeling software, such as the biomechanical simulation software called Anybody [2].

In recent years, many eye movement models have been developed [1, 5–13]. Eye movement modeling depends on the mechanical properties of six EOMs and those of the peripheral orbital suspension tissues. Current studies mainly focus on EOMs, while the study of orbital tissue is not precise enough. In the famous Orbit™ 1.8 gaze mechanics simulation, orbital suspension tissues were modeled as a homogeneous spring that exerts a recentering force [7–9]. Priamikov et al. presented two eye movement models and integrated them into the available musculoskeletal models [1]. Iskander et al. introduced OpenEyeSim as a platform for developing models of eye movement control in the perception–action cycle [2]. The stiffness of the above models established by Priamikov (5 mN/°) and by Iskander (4.8 mN/°) came from the experimental data of Robinson et al. in whose experiment the stiffness of orbital suspension tissues was obtained during horizontal eye rotation with both horizontal recti detached [5]. In our previous models, orbital suspension tissues were also greatly simplified to an integrated resistance moment [14, 15]. It is generally believed that the resistance moment is linear to the angle of the eyeball and that the coefficient is the limiting stiffness of orbital suspension tissues. For stiffness, existing data range from 1 to 10 mN/° [1, 2, 5–9], and there is not a unified reference. To date, the accuracy of eye movement models is not high enough because the influence of EOMs on orbital suspension tissues has not been excluded in previous experimental studies on orbital suspension tissues [5, 16–18].

The resistance and stiffness of orbital suspension tissues with four or six EOMs attached to the eyeball has been studied by different investigators using in vivo experiments [5, 16–18]. In previous studies, investigators studied the resistance and stiffness of orbital suspension tissues without excluding the effect of EOMs, possibly because of the complex anatomy and ethical limitations. The mechanical behavior of orbital suspension tissues without EOMs is absolutely necessary to establish an accurate eye movement model because modeling studies have shown that orbital suspension tissues play an indispensable role in supporting the eyeball and stabilizing the movement path of EOMs during eye movement [2, 19, 20]. Only a few works in the literature have reported on the differences in the resistance and stiffness of orbital suspension tissues, whether synthetically considering EOMs or not. The present work aims to study the resistance and stiffness of orbital suspension tissues with or without EOMs using an animal experiment in vivo.

Rabbits are usually used as experimental animals in basic experiments. The body of a rabbit is small, and its eyeball is relatively large. The rabbit orbit is remarkably more complete in comparison with that of many other lower animals, although the rabbit orbit is not better than the human orbit from an evolutionary perspective, and little fat is present in the rabbit orbit except for small masses attached to glands and muscles [21]. Although the anatomy of the rabbit orbit is not the same as that of the human orbit, human and rabbit eyes have numerous common characteristics, such as the eye movements of rabbits and of humans being controlled by six EOMs and orbital suspension tissues [21]. In this study, New Zealand white (NZW) rabbits were selected as an experimental animal model. The resistance and stiffness of orbital suspension tissues with four EOMs were compared with those without EOMs, and the differences were researched. Then, the influence of the EOMs on orbital suspension tissues was clarified and it can be used to improve the accuracy of eye movement model. The results of this study may provide a reference for further investigations of human orbital tissues.

## Materials and methods

### Animals and anesthesia

The in vivo experiments were conducted according to the Association for Research in Vision and Ophthalmology (ARVO) Statement for the use of Animals in Ophthalmic and Vision Research. Without considering gender, fifteen healthy adult New Zealand white (NZW) rabbits (2.41 ± 0.13 kg) were used in the experiment. Prior to the experiment, rabbits were under general anesthesia. Rabbits were intraperitoneally injected with 35 ml of a chloral hydrate mixture (provided by Bethune International Peace Hospital of PLA). If a rabbit woke up on the lab bench, another 5 ml of the chloral hydrate mixture was reinjected. During the experiment, Alcaine (2 drops/min) was applied to the tested eyeball to keep the rabbit eye moist.

### Experimental design

The fifteen rabbits were marked as no. 1–15. The resistance of the orbital tissues of rabbit no. 1 was not considered due to the corresponding EOMs being damaged during surgery. The experiment included four cases: (1) the two horizontal recti (LR and MR) were detached from the left eye; (2) six EOMs were detached from the left eye; (3) the two vertical recti (SR and IR) were detached from the right eye; and (4) six EOMs were detached from the right eye. During the experiment, the left eye was moved horizontally in the horizontal recti acting plane. The right eye was moved vertically in the vertical recti acting plane.

### Experimental procedures

**Fig. 1:**
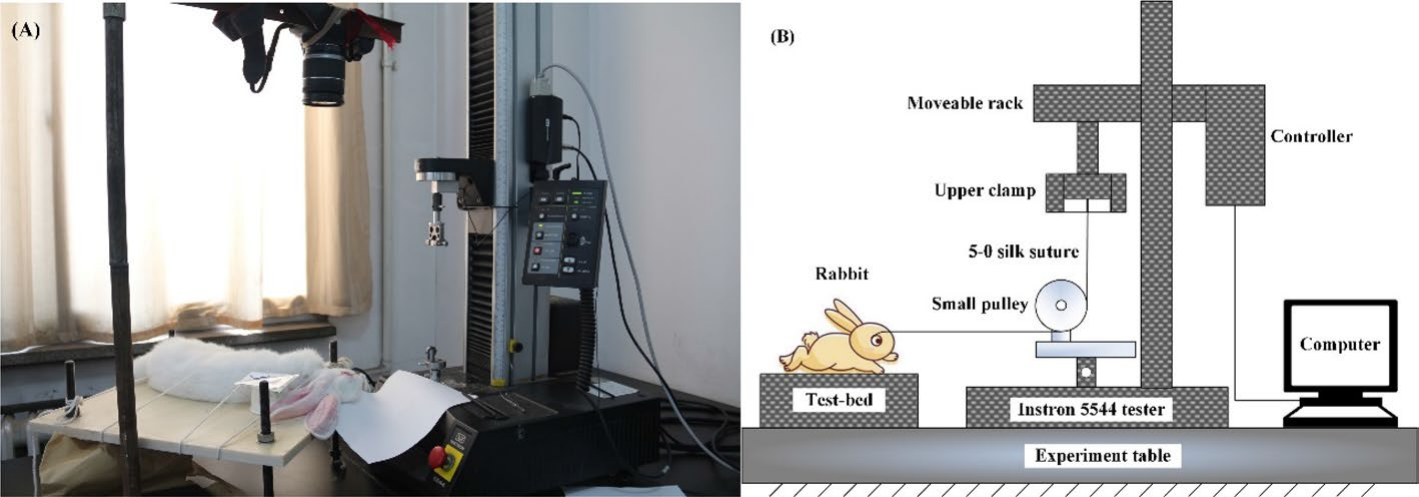
Experimental setup. **a** Picture of real products; **b** schematic diagram

Prior to mechanical testing, a rabbit was operated on while under general anesthesia and on a test-bed (Fig. 1). The blepharostat was used to uncover the conjunctiva and fascia from the EOMs, and then, the insertions of six EOMs were exposed. The two horizontal recti (i.e., LR and MR) of the left eye were detached from the insertions on the eyeball. Then, two 5-0 silk sutures were seamed to the residual insertions on the isolated eyeball. After the operation, the height of the test-bed was adjusted to match the posture of the rabbit to upturn the corneal center.

As shown in Fig. 1, the height of the corneal center was ascertained to be at the same level as the lowest point of the pulley, and then, the suture was tangentially seamed to the eyeball. The free end of the suture was fixed to the upper clamp of the tester. The resistance of the orbital suspension tissues was determined by a tester (Instron 5544, Instron Co. Ltd., Norwood, Colorado, USA) with a 5.0 N full scale load cell (accuracy 2‰). A set of data was produced every 0.1 s, and approximately 650 sets of data were obtained for each sample. The stretching velocity was set as 5 mm/min, which is equivalent to the eyeball rotating with an angular velocity of 0.5 °/s. However, the angular velocity of both fixations and rapid eye movements can be up to 900 °/s [12]. The test was stopped when the tensile displacement reached 5 mm with the rabbit eyeball rotated nearly 30° within the physiological range [17]. After unloading, the other four EOMs were detached from the left eye and the two seamed sutures were reserved. The similar tensile test was re-employed on the two sutures. The corresponding data of the load (N) and the displacement (mm) were recorded by a computer.

After the operation of the left eye was completed, the right eye was operated on. Two vertical recti (i.e., SR and IR) were detached from the insertions on the eyeball, and then, two 5-0 silk sutures were seamed to the stumps of the eyeball. The remaining operation steps for the right eye were according to those that were used for the left eye. The difference was that the left eye moved horizontally while the right eye moved vertically during the tensile test. After the tensile test, the size of the eyeball was measured by a triangular ruler and a camera in vitro. Image-Pro 5.1.2C was used to read the size of the eyeball in three dimensions.

### Statistical analysis

All results were reported as mean ± standard deviation and statistically performed by using SPSS v.17.0 software and one-way analysis of variance (ANOVA) analysis. A *P* value < 0.05 was considered to indicate a statistically significant difference.

## Results

### Eyeball size

**Fig. 2:**
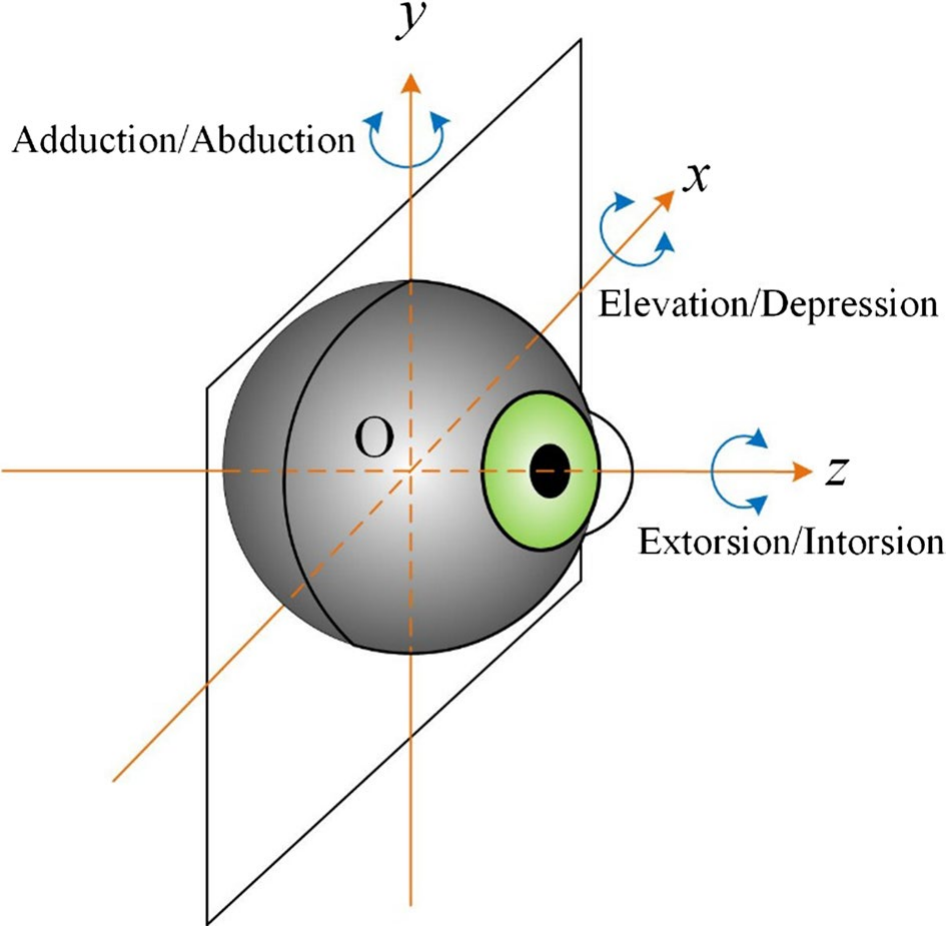
O*xyz* coordinate system of the eyeball

During the tensile test, the displacement of the suture was equal to the arc length of eye rotation. The radius of the left eyeball in the *x*O*z* plane (Fig. 2) was the mean value of the radii between the *x*- and *z*-axes, i.e., *r *= (*x* + *z*)/2 (Table 1). The radius of the right eyeball in the *y*O*z* plane (Fig. 2) was the mean value between the radii along the *y*- and *z*-axes, i.e., *r* = (*y* + *z*)/2 (Table 1). The relationship between the arc length and angle of eye rotation is as follows:

1 $$ \theta \,{ = }\,\,\left( {{s \mathord{\left/ {\vphantom {s r}} \right. \kern-0pt} r}} \right) *\left( {{{ 1 8 0} \mathord{\left/ {\vphantom {{ 1 8 0} \pi }} \right. \kern-0pt} \pi }} \right), $$

where *θ* is the angle of eye rotation (°), *s* is the arc length of eye rotation (i.e., the displacement of the suture in the tensile test; mm), and *r* is the radius of the eyeball (mm). Table 1 and Eq. (1) indicate that a 1 mm arc length corresponded to 6.31° during horizontal left eye movement, while a 1 mm arc length corresponded to 6.37° during vertical right eye movement.

**Table 1:** Radii of the eyes of 15 rabbits

	Radius of the left eye (mm)				Radius of the right eye (mm)			
	*x*	*y*	*z*	(*x *+ *z*)/2	*x*	*y*	*z*	(*y *+ *z*)/2
Mean	9.34	9.13	8.83	9.09	9.54	9.12	8.89	9.01
Standard deviation	0.32	0.28	0.35	0.27	0.21	0.23	0.22	0.17

### Resistance of orbital suspension tissues

**Fig. 3:**
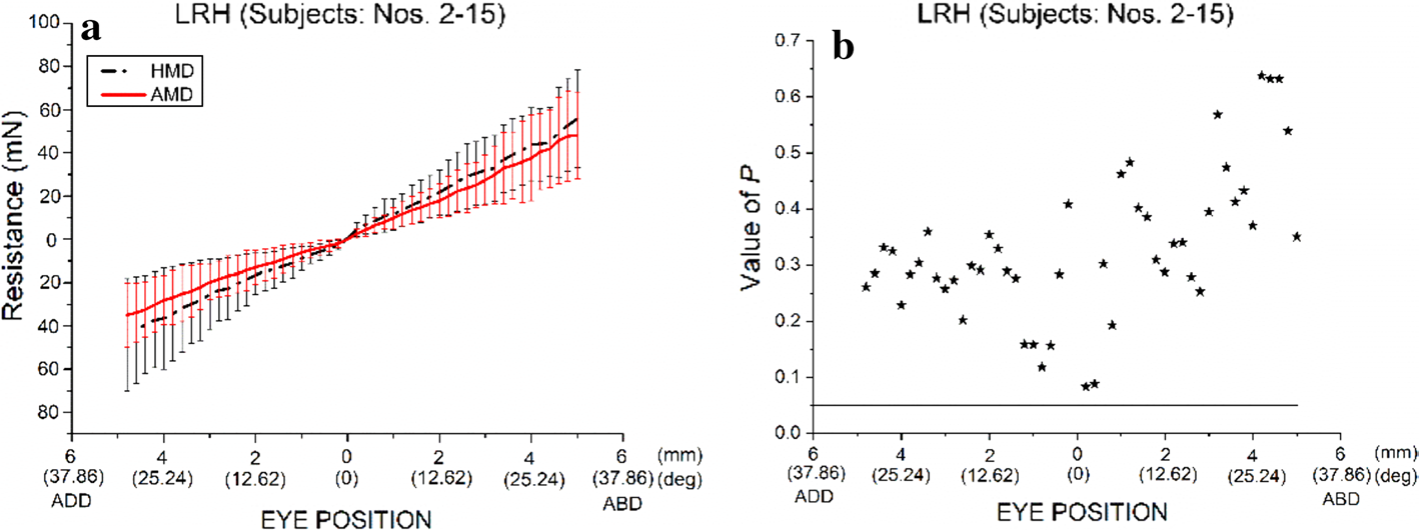
Comparison of the resistance in orbital suspension tissues with the left eyes of rabbit nos. 2–15 rotating horizontally (LRH) between the two cases [the two horizontal muscles are detached (HMD) in the first case, whereas all EOMs are detached (AMD) in the second case]. **a** Eye position–resistance relationship. **b** Statistical differences in the resistances of orbital suspension tissues between the two cases

When the left eye rotated horizontally, with LR and MR detached, the orbital suspension tissues mainly resisted the external force driving the eye movement, and the other four EOMs played a small role. The eye position–resistance relationships of the orbital suspension tissues with the left eye of rabbit nos. 2–15 rotated horizontally are shown in Fig. 3a. The statistical differences in the resistances of orbital suspension tissues between when the two horizontal EOMs were detached (HMD) and when all EOMs were detached (AMD) are shown in Fig. 3b. The statistical differences refer to the differences in the resistances between these two cases when the eyes were located in different eye positions. At each certain eye position (such as the position that the eye rotates an arc of 1 mm horizontally), the resistances (14 sample values) of HMD were compared with the resistances (14 sample values) of AMD. The statistical difference is represented by a *P* value. The resistances of HMD were not significantly different (*P* > 0.05) from those of AMD. When the eye was rotated temporally (abducted) from 0° (*s* = 0 mm) to 31.55° (*s* = 5 mm), the resistance of HMD increased almost linearly from 0 to 55.81 mN and the resistance of AMD increased almost linearly from 0 to 48.06 mN. When the eye was rotated nasally (adducted) from 0° (*s *= 0 mm) to 31.55° (*s* = 5 mm), the resistance of HMD increased almost linearly from 0 to 44.08 mN and that of AMD increased almost linearly from 0 to 34.95 mN (Fig. 3b).

**Fig. 4:**
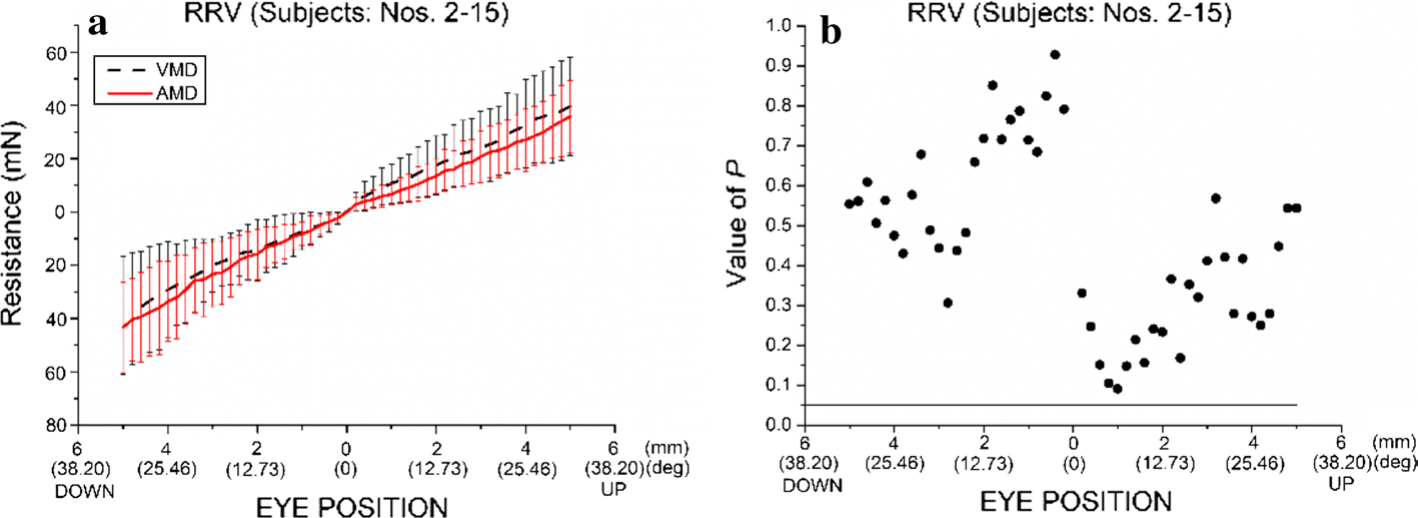
Comparison of the resistance of the orbital suspension tissues of the right eyes of rabbits nos. 2–15 rotating vertically (RRV) between two cases [the two vertical muscles are detached (VMD) in the first case, whereas all EOMs are detached (AMD) in the second case]. **a** Eye position–resistance relationship. **b** Statistical differences in the resistances of orbital suspension tissues between the two cases

When the right eye was rotated vertically, with SR and IR detached, the orbital suspension tissues primarily resisted the external force driving the eye movement, which was contrary to the small role of the other four EOMs. The eye position–resistance relationships of the orbital suspension tissues with the right eyes of rabbit nos. 2–15 rotated vertically are shown in Fig. 4a. The statistical differences in the resistances of the orbital suspension tissues between the case of the two vertical EOMs detached (VMD) and that of AMD are shown in Fig. 4b. The statistical differences represent the differences of resistances between these two cases when the eye was located in different eye positions. At each certain eye position (such as the position at which the eye rotated an arc of 1 mm vertically), the resistances (14 sample values) of VMD were compared with the resistances (14 sample values) of AMD. Statistically significant differences are represented by a *P* value. The resistances (Fig. 4b) of VMD and those of AMD were not significantly different (*P *> 0.05). When the eye was rotated upward from 0° (*s* = 0 mm) to 31.83° (*s* = 5 mm), the resistance of VMD increased almost linearly from 0 to 39.64 mN and that of group AMD increased almost linearly from 0 to 35.85 mN. When the eye was rotated downward from 0° (*s* = 0 mm) to 31.83° (*s* = 5 mm), the resistance of the VMD increased almost linearly from 0 to 38.76 mN and that of AMD increased almost linearly from 0 to 43.22 mN.

### Stiffness of orbital suspension tissues

**Fig. 5:**
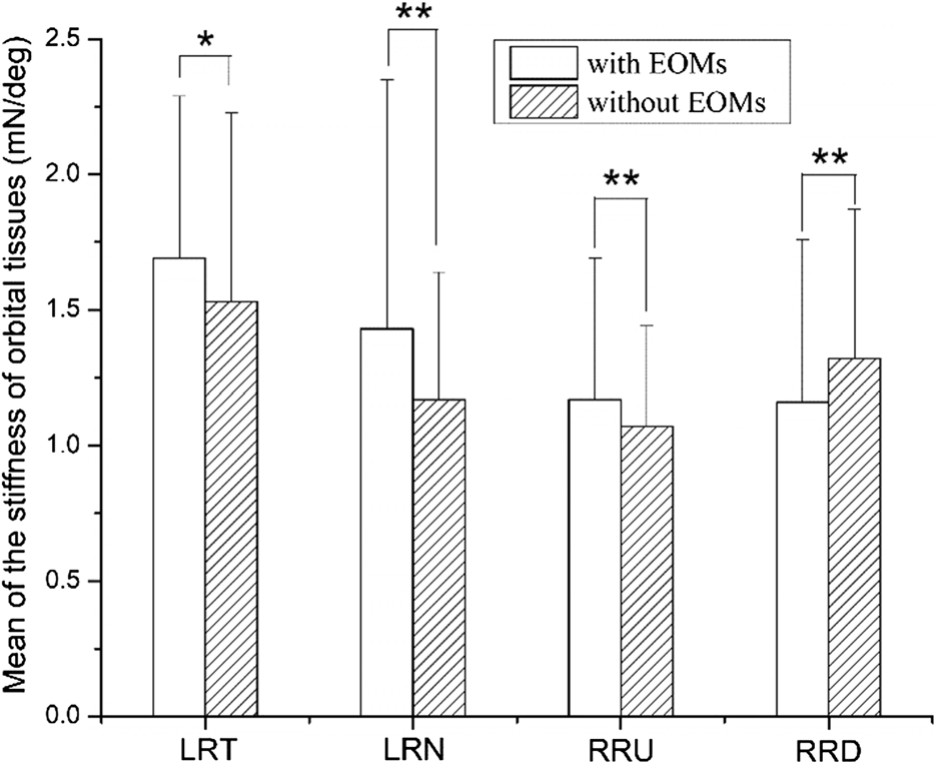
Statistical analysis of the stiffness between orbital suspension tissues with EOMs and without EOMs. LRT, the left eye rotating temporally; LRN, the left eye rotating nasally; RRU, the right eye rotating upward; RRD, the right eye rotating downward. *Denotes statistical differences (*P *< 0.05), and **denotes significant statistical differences (*P *< 0.01)

The resistance of orbital suspension tissues varied almost linearly with the eye rotation (Figs. 3a, 4a). Therefore, the stiffness of each sample of orbital suspension tissue was obtained by linear fitting the data of the eye position–resistance of each sample for rabbit nos. 2–15 in eight different cases, and the *P* values of the linear fitting were nearly zero. The mean ± standard deviations for the stiffness are presented in Table 2. The mean values of the stiffness vary within the range of 1–1.7 mN/°. The statistical analysis of the stiffness was obtained by comparing the stiffness of orbital suspension tissues with EOMs and those without EOMs (Table 2). The comparative results of the stiffness show that they are significantly different between orbital suspension tissues with EOMs and those without EOMs (Fig. 5). The stiffness of orbital suspension tissues with EOMs statistically differed from those without EOMs when the left eye rotated temporally (LRT) (*P *= 0.022). The stiffness of orbital suspension tissues with EOMs was significantly different from those without EOMs when the left eye rotated nasally (LRN) (*P *= 0.004), the right eye rotated upward (RRU) (*P *= 0), and the right eye rotated downward (RRD) (*P* = 0.004).

**Table 2:** Stiffness of the orbital suspension tissues of rabbit nos. 2–15

Sample no	LRT-HMD (mN/°)	LRT-AMD (mN/°)	LRN-HMD (mN/°)	LRN-AMD (mN/°)	RRU-VMD (mN/°)	RRU-AMD (mN/°)	RRD-VMD (mN/°)	RRD-AMD (mN/°)
2	2.47	3.22	3.71	1.78	1.65	1.60	2.69	1.46
3	2.99	1.49	1.31	1.02	0.66	0.45	0.96	1.26
4	2.07	1.48	1.00	1.04	1.29	1.33	0.72	0.99
5	1.23	1.03	1.90	1.91	2.61	1.80	2.17	2.79
6	1.89	1.48	1.24	0.99	0.87	0.70	0.78	0.95
7	2.04	2.92	2.92	2.10	1.15	1.22	0.76	1.40
8	1.25	1.51	1.39	1.09	1.02	0.76	1.14	1.75
9	1.91	1.41	0.53	0.36	1.33	1.05	0.69	0.98
10	1.33	1.03	0.86	0.77	1.14	1.08	0.98	1.00
11	0.88	1.22	0.38	0.88	1.22	1.30	1.62	2.10
12	1.28	1.62	1.59	1.10	0.69	0.91	0.64	1.04
13	1.37	0.69	1.09	1.33	1.34	1.10	1.17	1.01
14	1.01	1.14	0.55	0.92	0.52	0.76	0.93	0.98
15	1.87	1.18	1.55	1.15	0.85	0.86	0.93	0.83
M ± S	1.69 ± 0.60	1.53 ± 0.70	1.43 ± 0.92	1.17 ± 0.47	1.17 ± 0.52	1.07 ± 0.37	1.16 ± 0.60	1.32 ± 0.55

LRT, left eye rotating temporally; LRN, left eye rotating nasally; HMD, two horizontal EOMs detached; AMD, all EOMs detached; RRU, right eye rotating upward; RRD, right eye rotating downward; VMD, two vertical EOMs detached; and AMD, all EOMs detached; M ± S denotes the mean ± standard deviation

## Discussion

The resistance and stiffness of orbital suspension tissues are essential for accurate eye movement modeling. The influence of extraocular muscles on the resistance and stiffness of orbital suspension tissues was studied by using 15 NZW rabbits in this work. There is no significant difference between the resistance of orbital suspension tissues with four EOMs and those without EOMs (Figs. 3, 4). This result shows that the two horizontal/vertical recti play major roles in horizontal/vertical eye movement, consistent with the function of the EOMs [12]. According to the function of each extraocular muscle, the LR and MR share a common horizontal plane. Contractions of these two muscles produce horizontal eye movements. In other words, the other four extraocular muscles (i.e., SR, IR, SO, and IO) play a small role in the horizontal eye movement. The SR and IR muscles form the vertical agonist–antagonist pair, which mainly controls vertical eye movement [12]. In addition, the other four extraocular muscles (i.e., LR, MR, SO, IO) only play a small role in vertical eye movement. The objective of this study was to determine the influence of EOMs on the resistance and stiffness of orbital suspension tissues and to lay a foundation for accurate eye movement modeling.

The resistance and stiffness of orbital suspension tissues, which include six EOMs or four EOMs, have been reported in previous experimental studies [5, 16–18]. In this experiment, the stiffness of orbital suspension tissues in eight cases of eye movement was determined and is shown in Table 2. A summary of stiffness of the orbital suspension tissues is presented in Table 3 [16, 17, 22, 23]. In this work, the maximum resistance of orbital suspension tissues without EOMs was 48.06 mN when the left eye rotated horizontally and was 43.22 mN when right eye rotated vertically, (Figs. 3a and 4a, respectively). The corresponding results show that orbital suspension tissues without EOMs played an important role in eye movement, and this finding is consistent with previous experimental results [19, 20, 24, 25]. In eye movement modeling, the action of orbital suspension tissues is simplified to a comprehensive effect, namely, a total resistance moment. It is generally believed that the resistance moment is proportional to the angle of the eyeball and that the proportional coefficient is the stiffness of the orbital suspension tissues. In the existing model, the stiffness of orbital suspension tissues with EOMs was measured [5, 16–18]. Because of a lack of experimental data, such inaccurate stiffness data have to be used in modeling [2, 16, 17, 19, 26]. However, we found that there was a significant difference between the stiffness of orbital suspension tissues with EOMs and those without EOMs (Fig. 5). Therefore, the stiffness data with EOMs cannot be used to establish the eye movement model directly.

**Table 3:** Comparison between different stiffness of the orbital suspension tissues in the literature and this work

Experiment	Description	Stiffness (mN/°)	EOMs
Collins [16]	In primary innervation when the eye rotated nasally from 0° to 45° (human)	4.91	With LR and MR detached
	In primary innervation when the eye rotated temporally from 0° to 45° (human)	9.82	With LR and MR detached
Collins et al. [17]	The eye rotated with a nasal innervation of 30° (human)	10.30	With all EOMs attached
	The eye rotated with a temporal innervation of 30° (human)	9.22	With all EOMs attached
Scott [22]	The eye had a horizontal eye movement of 40° (human)	4.91	With the LR and MR detached
Barmack [23]	The eye moved horizontally or vertically to 35° (rabbit)	1.08 ± 0.29	With six EOMs attached
This work	The eye rotated temporally from 0° to 31.55° (rabbit)	1.69 ± 0.60	With LR and MR detached
		1.53 ± 0.70	With all EOMs detached
	The eye rotated nasally from 0° to 31.55° (rabbit)	1.43 ± 0.92	With LR and MR detached
		1.17 ± 0.47	With all EOMs detached
	The eye rotated upward from 0° to 31.83° (rabbit)	1.17 ± 0.52	With SR and IR detached
		1.07 ± 0.37	With all EOMs detached
	The eye rotated downward from 0° to 31.83° (rabbit)	1.16 ± 0.60	With SR and IR detached
		1.32 ± 0.55	With all EOMs detached

Stiffness was significantly different in the two tested conditions, but resistance was not, possibly because resistance is determined by data points and stiffness is determined by the slope of the straight line of the linear fit of the corresponding resistance data points. Stiffness data can be directly used in eye movement modeling. A limitation of this study is the individual differences among the NZW rabbits, which may have led to some errors in the results. Another limitation of this study is the effects of the small pulley on the experimental setup. In this experiment, the tension of the suture varied over a small range so that the friction of the small pulley also varied over a small range. Eliminating the effects of uniform friction is challenging. The resistance of the pulley was ignored in the load results during the test. In addition, the stiffness value is not a constitutive property of any particular biological material but is only a gross lumped parameter that is operationally defined and is suitable for only certain types of ocular motor models. This work only lays a foundation for studying the effect of human orbital suspension tissues.

## Conclusions

In summary, the two horizontal recti and the two vertical recti play important roles in passive horizontal eye movement and in passive vertical eye movement, respectively. We determined the influence of the EOMs on orbital suspension tissues. In addition, we discovered that the stiffness of orbital suspension tissues with EOMs was not appropriate for use in the horizontal and vertical eye movement model. We need to further study whether the stiffness of orbital suspension tissues without EOMs can be replaced by the stiffness of orbital suspension tissues with EOMs in other types of eye movement. The next phase of our research will establish an eye movement model using the stiffness of orbital suspension tissues without EOMs. These results will provide further theoretical guidance for the precise determination of the surgical amount of EOMs.

## Data Availability

All data used and analyzed during the current study available from the corresponding author on reasonable request.

## References

[1] Priamikov A, Fronius M, Shi B, Triesch J (2016). OpenEyeSim: a biomechanical model for simulation of closedloop visual perception. J Vision..

[2] Iskander J, Hossny M, Nahavandi S, del Porto L (2018). An ocular biomechanic model for dynamic simulation of different eye movements. J Biomech.

[3] Mckean-Cowdin R, Cotter SA, Tarczy-Hornoch K, Wen G, Kim J, Borchert M, Varma R (2013). Prevalence of amblyopia or strabismus in Asian and non-hispanic white preschool children: multi-ethnic pediatric eye disease study. Ophthalmology.

[4] Kim EY, Roper-Hall G, Cruz OA (2016). Effectiveness of bilateral lateral rectus resection for residual esotropia in dysthyroid ophthalmopathy. J Aapos..

[5] Robinson DA, O’Meara DM, Scott AB, Collins CC (1969). Mechanical components of human eye movements. J Appl Physiol.

[6] Robinson DA (1975). A quantitative analysis of extraocular muscle cooperation and squint. Invest Ophthalmol..

[7] Miller JM, Robinson DA (1984). A model of the mechanics of binocular alignment. Comput Biomed Res.

[8] Miller JM, Pavlovski DS, Shamaeva I. Orbit™ 1.8 Gaze Mechanics Simulation, Eidactics, San Francisco. 1995.

[9] Miller JM. Orbit™ 1.8 Gaze mechanics simulation, Eidactics visual biosimulation, San Francisco. 1999; p. 1–160.

[10] Simonsz HJ, Spekreijse H (2009). Robinson’s computerized strabismus model comes of age. Strabismus..

[11] Pascolo P, Carniel R (2009). From time series analysis to a biomechanical multibody model of the human eye. Chaos Solitons Fract..

[12] Wei Q, Sueda S, Pai DK (2010). Physically-based modeling and simulation of extraocular muscles. Prog Biophys Mol Bio..

[13] Iskander J, Hossny M, Nahavandi S (2018). A review on ocular biomechanic models for assessing visual fatigue in virtual reality. IEEE Access..

[14] Gao ZP, Guo HM, Chen WY (2014). Initial tension of the human extraocular muscles in the primary eye position. J Theor Biol.

[15] Guo HM, Gao ZP, Chen WY (2016). The biomechanical significance of pulley on binocular vision. Biomed Eng Online..

[16] Collins CC, Bach-Y-Rita P, Collins CC, Hyde JE (1971). Orbital mechanics. The control of eye movements.

[17] Collins CC, Carlson MR, Scott AB, Jampolsky A (1981). Extraocular muscle forces in normal human subjects. Invest Ophthalmol Vis Sci.

[18] Simonsz HJ, Crone RA, de Waal BJ, Schooneman M, de Haas HA (1984). Measurement of the mechanical stiffness in cyclotorsion of the human eye. Vision Res.

[19] Schutte S, van den Bedem SP, van Keulen F, van der Helm FC, Simonsz HJ (2006). A finite-element analysis model of orbital biomechanics. Vision Res.

[20] Karami A, Eghtesad M, Haghpanah SA (2017). Prediction of muscle activation for an eye movement with finite element modeling. Comput Biol Med.

[21] Davis FA (1929). The anatomy and histology of the eye and orbit of the rabbit. Trans Am Ophthalmol Soc.

[22] Scott AB, Bach-Y-Rita P, Collins CC, Hyde JE (1971). Extraocular muscle forces in strabismus. The control of eye movements.

[23] Barmack NH (1976). Measurements of stiffness of extraocular muscles of the rabbit. J Neurophysiol.

[24] Bron AJ (1997). “Wolf’s anatomy of the eye and orbit”, Chapter 4. The extraocular muscles and ocular movements.

[25] Cirovic S, Bhola RM, Hose DR, Howard IC, Lawford PV, Parsons MA (2005). A computational study of the passive mechanics of eye restraint during head impact trauma. Comput Methods Biomech Biomed Engin..

[26] Guo HM, Gao ZP, Chen WY (2016). Contractile force of human extraocular muscle: a theoretical analysis. Appl Bionics Biomech..

